# An Infrastructure-Free Magnetic-Based Indoor Positioning System with Deep Learning

**DOI:** 10.3390/s20226664

**Published:** 2020-11-20

**Authors:** Letícia Fernandes, Sara Santos, Marília Barandas, Duarte Folgado, Ricardo Leonardo, Ricardo Santos, André Carreiro, Hugo Gamboa

**Affiliations:** 1 Associação Fraunhofer Portugal Research, Rua Alfredo Allen 455/461, 4200-135 Porto, Portugal; sara.santos@fraunhofer.pt (S.S.); marilia.barandas@fraunhofer.pt (M.B.); duarte.folgado@fraunhofer.pt (D.F.); ricardo.leonardo@fraunhofer.pt (R.L.); ricardo.santos@fraunhofer.pt (R.S.); andre.carreiro@fraunhofer.pt (A.C.); hugo.gamboa@fraunhofer.pt (H.G.); 2 Laboratório de Instrumentação, Engenharia Biomédica e Física da Radiação (LIBPhys-UNL), Departamento de Física, Faculdade de Ciências e Tecnologia, FCT, Universidade Nova de Lisboa, 2829-516 Caparica, Portugal

**Keywords:** indoor positioning systems, infrastructure-free, magnetic field, deep neural networks, smartphones, fingerprinting

## Abstract

Infrastructure-free Indoor Positioning Systems (IPS) are becoming popular due to their scalability and a wide range of applications. Such systems often rely on deployed Wi-Fi networks. However, their usability may be compromised, either due to scanning restrictions from recent Android versions or the proliferation of 5G technology. This raises the need for new infrastructure-free IPS independent of Wi-Fi networks. In this paper, we propose the use of magnetic field data for IPS, through Deep Neural Networks (DNN). Firstly, a dataset of human indoor trajectories was collected with different smartphones. Afterwards, a magnetic fingerprint was constructed and relevant features were extracted to train a DNN that returns a probability map of a user’s location. Finally, two postprocessing methods were applied to obtain the most probable location regions. We asserted the performance of our solution against a test dataset, which produced a Success Rate of around 80%. We believe that these results are competitive for an IPS based on a single sensing source. Moreover, the magnetic field can be used as an additional information layer to increase the robustness and redundancy of current multi-source IPS.

## 1. Introduction

The improvements in Indoor Positioning Systems (IPS) technology have greatly impacted localisation-based services, consequently increasing their popularity and number of users [1]. Moreover, IPS have a large number of applications, such as navigation in stations and airports, emergency response services, guides in museums and shopping centres, and localisation-based advertising [1,2].

To overcome the poor performance of the Global Navigation Satellite System (GNSS) in indoor environments, several authors propose different IPS [3,4]. They usually resort to technologies such as the Radio Frequency Identification (RFID), Wi-Fi, Bluetooth, or Ultra-Wide Band (UWB) [5,6]. However, these technologies still present limitations that compromise the scalability of location-based services, as they require additional dedicated infrastructure [5]. For example, RFID requires tags, UWB uses anchors, and Bluetooth searches for beacons at various landmarks to locate the users [7]. Although Wi-Fi is available in most buildings, this source lacks performance as its Radio Frequency (RF) signal suffers from shadowing and multi-path problems [7,8]. Furthermore, RF signals can cause electromagnetic interference with critical systems, forbidding their use in some locations, such as planes or hospitals.

On the other hand, infrastructure-free solutions dismiss the need for additional infrastructure. Fingerprinting is one of such techniques, which often leverages the pervasively available geomagnetic field and the Wi-Fi networks. It uses fingerprints, which are maps of the buildings with the annotation of the expected sensor reading at each location. They can then be compared to new data, to localise a user [9]. In general, fingerprinting-based solutions require an extensive process to collect and update buildings information, hindering their implementation in large-scale environments [10]. Moreover, these IPS can either be based on a single source, as the Wi-Fi signals, or on the combination of different sources of information, applying probabilistic methods such as particle filters. Still, the combination of different sources depends on Wi-Fi availability for the system’s initialisation. This Wi-Fi dependence is expected to become a major limitation for existing IPS, following recent scanning restrictions from Android devices (https://developer.android.com/guide/topics/connectivity/wifi-scan). Moreover, as the next generation of mobile networking technology, i.e., 5G, covers both the inside and outside of buildings, Wi-Fi may suffer a drop in usage. Therefore, an alternative solution that works in the absence of Wi-Fi is necessary.

We propose to replace the current dependency on Wi-Fi scans in infrastructure-free solutions by exploring the magnetic field inside buildings. In indoor environments, the magnetic field is highly disturbed by electromagnetic interference caused by ferromagnetic architectural features and electronic equipment. These disturbances create unique patterns that can be utilised for localisation. Some solutions have been developed concerning the use of the magnetic data in this context [5,7]. Magnetic field-based IPS often offer satisfactory levels of accuracy. Nevertheless, their performance may be compromised by technical considerations. Manufacturers install magnetometers with different characteristics in the devices, e.g., level of noise tolerance and sensitivity [7], which affect the collected signals. Moreover, additional issues might arise as the same magnetic field value can be measured at multiple locations [11].

We intend to overcome the limitations of magnetic field-based methods by providing more precise information over time. The proposed system is independent of the user and the device, as it does not solely rely on the absolute values of the magnetic field. We employ fingerprinting-based methods, together with data augmentation, to reduce the extensive data collection for fingerprinting.

**Figure 1:**
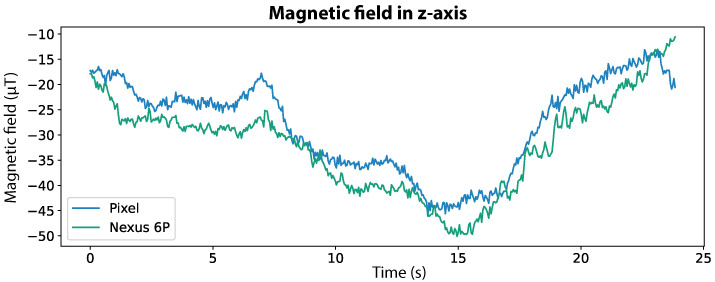
Magnetic field signals of the same trajectory perceived in the z-axis by two different smartphones: Pixel and Nexus 6P depicted in blue and green, respectively.

This novel methodology also leverages the potential of Deep Neural Networks (DNN), to replace the current dependence on Wi-Fi. Although the proposed system relies on the magnetic field only, our motivation is to extend its use to multi-source systems, either as an alternative method for the systems’ initialisation or to improve the positioning accuracy over time. For this study, we collected data with different smartphone models, to avoid device-specific dependency. Moreover, we based our work on the hypothesis that although consecutive magnetic field values may vary between two devices, the differences along a path remain similar, as illustrated in Figure 1, which shows the magnetic field measured by two different smartphone models. It can be noticed that although the absolute values are distinct, the signal shape is very similar.

As such, we propose to extract different features from magnetic sequences: (1) magnetic absolute values, (2) magnetic differences and (3) combinatorial magnetic differences. These features significantly reduce the ambiguity of magnetic data reported in indoor environments [2]. We use a DNN to estimate, given a new magnetic field reading, the corresponding probability of matching the correct position on a magnetic map. Then, two postprocessing techniques, one based on a threshold and the other based on the highest probability pixels, we produce the most probable regions for the user position.

The proposed indoor localisation methodology explores DNN capacities and magnetic features, providing an alternative for typical Wi-Fi-based approaches. Herewith, our major contributions are the following:

- An infrastructure-free indoor localisation system that relies on magnetic data and is robust to the sensors, variability between smartphones.
- A novel data augmentation algorithm that infers all possible magnetic sequences, within the building’s magnetic fingerprint, with only a set of predefined collected trajectories.
- A DNN architecture that is able to train different networks in each epoch, to be then merged into a consolidated output.
- Two different postprocessing methods to evaluate the performance of the magnetic field-based localisation system.

This paper is organised as follows: In Section 2, the related research on magnetic field-based IPS is discussed. In Section 3, the proposed method is described. In Section 4, the performance of our solution is presented and discussed, and in Section 5 the main conclusions are drawn and the future work envisioned.

## 2. Related Work

Magnetic field data has been explored by some authors, as an alternative source for IPS. RF-based localisation methods may be infeasible in large-scale scenarios, since the signal becomes more unstable as the buildings’ dimensions increase [11].

Often, particle filters are employed in the context of magnetic field-based IPS to estimate the user’s consecutive positions. Kim and Seo [4] proposed a system that contained both a particle filter and an encoder system. The particles’ weights were updated using multiple magnetic sensors and also three magnetic field maps, i.e. horizontal and vertical intensity maps, and a direction information map. Furthermore, in the propagation step, the particles were expanded by the velocity of the encoder. Some researchers focused on providing solutions to known issues related to magnetic data. For instance, the work proposed in [7] used magnetic data from several smartphones, creating Magnetic Patterns (MP) to overcome the limitation associated with different characteristics of magnetometers embedded in smartphones. The results showed an increased performance when compared to other MP approaches. Additionally, in the same study, an effort was also directed into analysing the impact in the localisation accuracy using distance metrics, such as the Euclidean distance and the Dynamic Time Warping (DTW) to match the MP. Contrarily, in our framework, we leverage DNN to learn the magnetic field patterns.

DNNs have been used in Wi-Fi-based indoor localisation [12,13]. However, few works report their use on magnetic field data. Lee and Han [2] propose a magnetic approach for indoor positioning using DNN to classify magnetic landmarks. In this study, recurrence plots were used as the input for the DNN, composed of convolutional and fully connected layers. While performing magnetic landmark classification, the authors also discovered that the magnetic data in the *z* axis was the most accurate when compared to xyz and xy alternatives. In the same context, an IPS called AMID was proposed in [8], which locates by recognising magnetic sequence patterns using a DNN. The authors remarked that the positioning error was related to the magnetic map resolution. Bae and Choe [11] presented a magnetic field-based IPS relying on Recurrent Neural Networks (RNN). The proposed method was compared with RF fingerprinting techniques showing better average positioning errors. However, for the experimental set-up, the authors used the magnetometer of a single smartphone to collect data, which may harm the generalisation to other devices.

The fusion of different sources has also emerged to overcome their individual limitations. In [14], the magnetic data was fused with visual images, to continuously track the user on a per-step basis using a particle filter that updates the location estimation after each user step. Additionally, a convolutional network was also considered as an approach to extract features from the used images. Finally, in [15], the fusion of magnetic and light data, using the smartphone’s sensors, was explored. This IPS used DNN, specifically Long-Short-Term-Memory (LSTM) layers, and analysed the impact of different design parameters, such as the test data size, the number of considered layers, and hidden units.

Some works in the literature leverage DNN for indoor location, but only a few use them with the magnetic field. Although large datasets are required to train DNN, data augmentation techniques can mend this requirement. In our research, we explore DNN composed of convolutional and LSTM layers with magnetic field data to locate the users. Our approach can later be integrated into multi-source systems towards a more robust localisation.

## 3. Framework for Indoor Positioning Using Magnetic Field

**Figure 2:**
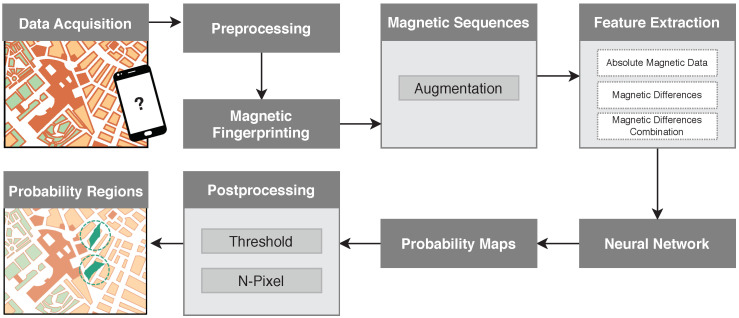
Pipeline of the proposed methodology.

This Section describes the proposed magnetic field-based IPS, which provides the most probable regions for the users’ location. The solution is depicted in Figure 2 and includes seven main stages: data acquisition, preprocessing, magnetic fingerprinting, magnetic sequences, feature extraction, neural network, and postprocessing.

In the first step, human trajectories were collected from the inertial sensors embedded in smartphones. In the preprocessing, the magnetic data is projected onto the Earth coordinates frame, for later use in the fingerprinting method. The created magnetic fingerprint allows defining several magnetic sequences. Additionally, more trajectories can be considered for building sequences through an implemented data augmentation algorithm. A feature extraction process retrieves the inputs for the DNN, which then outputs probability maps of possible locations. After postprocessing, our solution estimates the most probable regions for the specific users’ locations.

### 3.1. Preprocessing

Smartphones are typically equipped with a set of sensors, such as the Inertial Measurement Unit (IMU). IMUs incorporate an accelerometer, a gyroscope and a magnetometer, which collect data over three orthogonal axes.

The accelerometer is sensitive to both linear and gravitational accelerations, measured in m/s${}^{2}$. The gyroscope assesses the angular velocity in rad/s. At last, the magnetometer measures the strength of the local magnetic field in μT, which is composed of the geomagnetic and environmental fields.

As the readings from these sensors get severely affected by changes in the devices’ orientation and position, we apply the sensor fusion algorithm described in [16]. It translates the collected data to the global Earth frame, which is independent of the orientation and position of the device. A second-order complementary filter is applied, which takes into account the long-term reference of the gravity direction to the North, and the short-term accuracy of the gyroscope in measuring the angular rotation of the device. The collected signals were resampled to 100 Hz to remove the sampling rate differences between smartphones. Moreover, a second-order low pass filter with a cutting frequency of 1 Hz was applied to remove noise from signals.

By applying these preprocessing mechanisms to all collected data, we ensure that the proposed framework will work independently from the position and orientation of the devices.

### 3.2. Magnetic Fingerprinting

Buildings are characterised by multiple features, which relate in space to form a relatively unique, multi-dimensional feature spot map. The building map includes environmental features such as the Earth’s magnetic field fluctuations. Although the magnetic field remains naturally stable around the same area, its pattern is highly affected indoors. The presence of metallic construction materials and electrical equipment causes disturbances in the magnetic pattern, which commonly produce unique patterns that can be used to identify a specific location [10].

In this work, magnetic fingerprints were built with the traditional approach, where a user walks throughout the building while manually annotating reference points. The sensor fusion mechanism of Section 3.1 is applied to the data, and the temporal-dependence of values is eliminated by converting them to the spatial domain. This process stores location-dependent characteristics of the magnetic field signal into a multi-dimensional fingerprint, to provide an absolute reference for the localisation. The fingerprint is then characterised by a set of two-dimensional coordinates (x,y), in meters, associated with the magnetic field vector in the Earth frame $\left(v_{x},v_{y},v_{z}\right)$, in μT.

One major concern of fingerprinting-based solutions is the time-consuming data collection process, especially in large buildings. Different techniques that may be applied, ranging from point-by-point calibration to walking surveys between reference points with posterior interpolations and extrapolations. Nevertheless, this process does not limit our solution, as instead of searching for an exact position, we search for the most probable region for the user’s location. Thus, we dismiss the need to cover the buildings’ entire area during data collection.

### 3.3. Magnetic Sequences

The absolute localisation of a user using only the magnetic field is not trivial, as different locations inside a building may have similar magnetic field values. Hence, we cannot rely exclusively on the absolute value of the magnetic field in a specific location to determine the user’s position. On the other hand, a set of magnetic field values along a trajectory is more distinctively representative of the user’s final location. Moreover, different devices show different absolute values in the same locations, but when considering a sequence, the differences between consecutive values remain similar for the same trajectory.

Thus, each user trajectory is segmented on short sequences of length *L* based on the temporal magnetic field data conversion to the distance domain.

### 3.4. Data Augmentation

**Figure 3:**
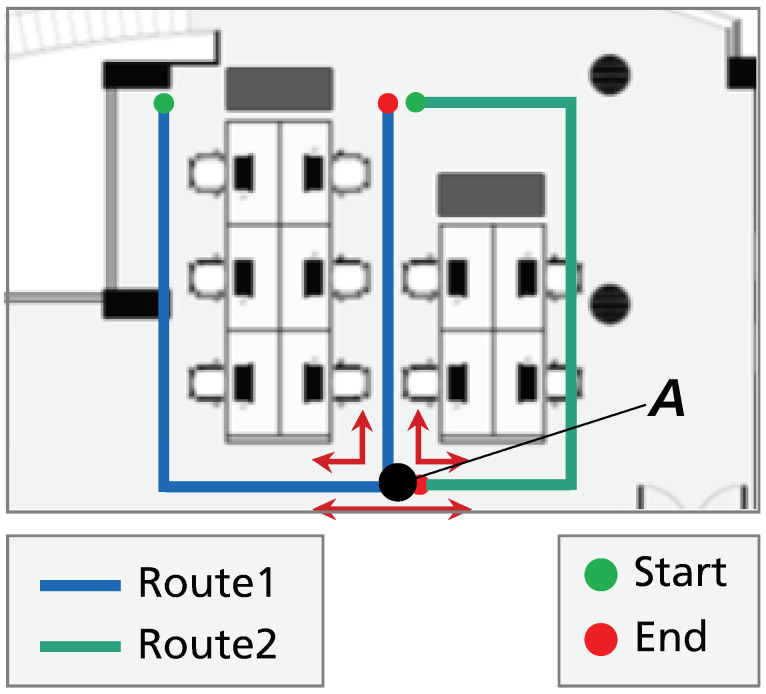
Data augmentation example for the training set. Route 1, in blue, and Route 2, in green, allow the definition of six different magnetic sequences from point A, represented by the red arrows.

To ease the data collection process, we designed a set of predefined trajectories to cover the entire building locations, but not all possible trajectories neither directions were recorded. Instead, we developed an augmentation algorithm that finds all possible trajectories of length *L* in the magnetic field fingerprint. The constructed fingerprint creates connections between some positions of the collected trajectories, which allows the extraction of trajectories that were not acquired during the data collection procedure and the extraction of trajectories in opposite directions. For instance, considering the two trajectories represented in Figure 3, the connections between Route 1 and Route 2 create artificial trajectories as represented in point A. Thus, the augmentation algorithm is able to retrieve six different magnetic sequences of length L at point *A*.

Moreover, to avoid overfitting to the fingerprint data, noise was added to the magnetic sequences in the data augmentation process. Two levels of random noise were applied from Normal distribution of mean 0 and variance 1, and then scaled by 0.1 and 0.2 multiplication factors, together with one level of random noise, retrieved from the Normal distribution of mean 0.02 and variance 0.2. The implemented data augmentation process is one of the key advantages of this work, as it greatly simplifies the effort necessary for collecting data. From this approach, it is possible to construct fingerprints with a limited set of trajectories without covering the entire space.

### 3.5. Feature Extraction

A magnetic field sequence is represented by a 4-dimensional vector given by $S=\{v^{\left(1\right)},\ldots ,v^{\left(L\right)}\}$ where $v^{\left(i\right)}$ denotes the *i*-th element in the set of the magnetic sequence values on the three orthogonal axes, (x,y,z), and magnitude, ∥m∥, and *L* represents the length of the magnetic sequence in pixels. An element *v* is represented by $v=\{v_{x},v_{y},v_{z},v_{∥m∥}\}$, where $v_{∥m∥}$ is given by Equation (1):

(1) $$v_{∥m∥}=\sqrt{{v_{x}}^{2}+{v_{y}}^{2}+{v_{z}}^{2}}$$

The feature set is composed of the magnetic absolute values, magnetic differences and combinatorial magnetic differences of the three orthogonal axes and magnitude. A description of each feature set is provided below. In Equations (2)–(4), $v_{ax}^{\left(i\right)}$ denotes the *i*-th element in the set of the magnetic sequence values on a specific ax component, where ax∈{x,y,z,∥m∥}.

Magnetic absolute values: The magnetic absolute values are the magnetic sequence values, with length *L*, without any transformation, given by the set in Equation (2).

(2) $$M_{ax}^{A}=\left\{v_{ax}^{\left(1\right)},\cdots ,v_{ax}^{\left(L\right)}\right\}$$

Magnetic differences: The magnetic differences correspond to the pixel-pixel difference along a magnetic sequence resulting in a set with L−1 length given by the set in Equation (3).

(3) $$M_{ax}^{D}=\left\{v_{ax}^{\left(2\right)}-v_{ax}^{\left(1\right)},\ldots ,v_{ax}^{\left(L\right)}-v_{ax}^{\left(L-1\right)}\right\}$$

Combinatorial magnetic differences: The combinatorial magnetic differences include all possible differences without repetition in a magnetic sequence resulting in a set with $\frac{L!}{2!(L-2)!}$ length given by Equation (4).

(4) $$M_{ax}^{C}=\left\{v_{ax}^{\left(2\right)}-v_{ax}^{\left(1\right)},v_{ax}^{\left(3\right)}-v_{ax}^{\left(1\right)},\ldots ,v_{ax}^{\left(L\right)}-v_{ax}^{\left(1\right)},v_{ax}^{\left(3\right)}-v_{ax}^{\left(2\right)},\ldots ,v_{ax}^{\left(L\right)}-v_{ax}^{\left(L-1\right)}\right\}$$

The feature set includes the magnetic differences and combinatorial magnetic differences, due to the variability of the absolute magnetic field values and different offset levels that are present in different smartphone models. The differences between magnetic field values are typically the same and are not influenced by different smartphones. The absolute values were also considered for the feature set as they can contain some meaningful information, apart from the differences, that can be extracted by deep learning techniques.

The labels used in this experiment are probability maps. For each prediction, we have a probability map with the same shape as the building map, where the user’s location is set with the highest probability (=1) and the remaining map is set to zero probability. The probability map sums to 1.

### 3.6. Deep Neural Network

Deep Learning techniques, as DNN, have been successfully applied in different contexts due to their strong ability to detect complex patterns. DNNs are composed of multiple non-linear functions mimicking the human brain. They can automatically learn features from higher levels of abstraction and complex functions that map the input to the output. Due to the complex nature of indoor localisation, DNNs are a competitive strategy for this application [17].

The DNN architecture proposed in this work contains the following layers:

- Convolutional Neural Network (CNN) layer: CNNs are commonly applied to image data, as they learn the two-dimensional representation of an image by successfully capturing spatial information through the application of filters. CNNs can be one-dimensional and be applied to time series, where they instead explore the temporal dependence between values.
- Long Short-Term Memory: LSTM is a type of RNN capable of learning long-term sequential or temporal dependencies [18].
- Flatten layer: A flatten layer converts a multidimensional input into a single dimension. This is usually used in the transition to a dense layer.
- Dense layer: A dense layer is a regular fully connected layer that densely connects all input neurons, from the previous layer, to all output neurons.
- Max-Pooling layer: A max-pooling layer is a down-sampling process that transforms the input, by reducing its dimension space, while storing the maximum value of (usually) non-overlapping regions.
- Dropout layer: A dropout layer, as the name suggests, drops out units in a neural network by randomly setting inputs units to zero, with a specified frequency, at each step in the training stage.

#### 3.6.1. Neural Network Architecture

**Figure 4:**
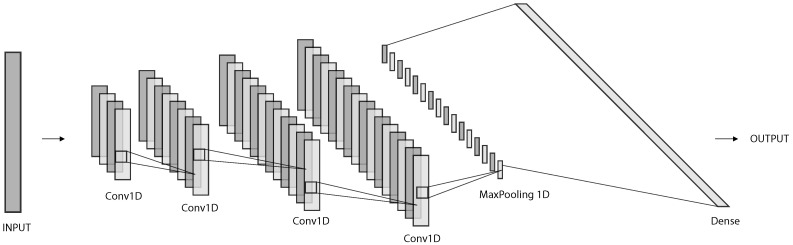
Neural Network Architecture for magnetic differences and combinatorial magnetic differences.

**Figure 5:**
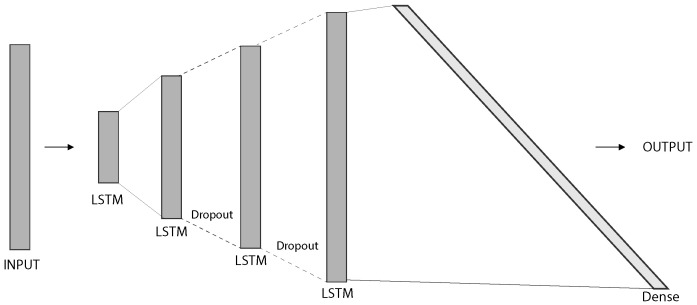
Neural Network Architecture for magnetic absolute values.

**Figure 6:**
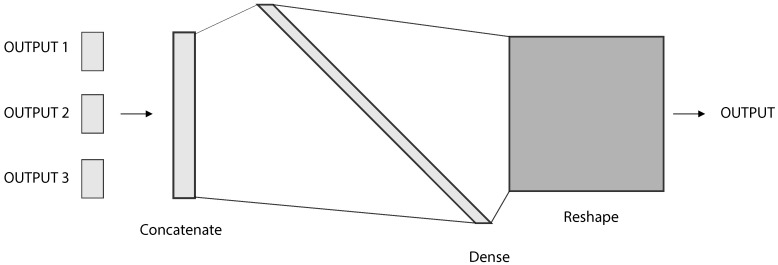
Illustration of the Neural Network Combination.

The neural network architecture used in this experiment is composed of four neural networks. The three sets of computed features have different lengths, so they were trained independently. In the end, a final network combines their results. For this process, three different types of architectures were used, namely DNN Type 1, Type 2, and a final Neural Network Combination. The illustrations in Figure 4, Figure 5 and Figure 6 were produced with the support of the NN-SVG Tool [19].

#### 3.6.2. DNN Type 1

The DNN Type 1 architecture was used twice, to learn both the magnetic differences and the combinatorial magnetic differences feature sets. This neural network is depicted in Figure 4 and contains four one-dimensional CNNs, followed by a one-dimensional max-pooling layer, a flatten layer, and a final dense layer. l1-l2 regularisation of factor 0.03 was applied to the third, fourth, and fifth layers, to prevent overfitting. Table 1 describes the designed layers’ details for this architecture.

#### 3.6.3. DNN Type 2

The DNN Type 2 was used to train the magnetic absolute values. Its architecture is depicted in Figure 5, and contains four LSTM layers, followed by one dense layer. Moreover, two dropout layers were introduced to reduce overfitting. The layers description is available in Table 2.

#### 3.6.4. Neural Network Combination

The final architecture aims to combine the individual results of the three individual trained networks. This network first concatenates the outputs of DNN Types 1 and 2. Then, a dense layer and a final reshape layer are applied to produce the output probability maps, as depicted in Figure 6. The dense layer description is available in Table 3.

#### 3.6.5. Neural Network Combination Loss

The training of neural networks uses loss functions to iteratively improve the prediction, by updating the weights of neurons. This update is done by subtracting to each weight their gradient with respect to the loss, depending on a predefined learning rate. In our approach, the Adam optimisation algorithm conducts this process, together with a categorical cross-entropy loss function, which is suitable for multi-class classification. The categorical cross-entropy loss quantifies the difference between two probability distributions, given by Equation (5):

(5) $$\sum_{i=1}^{N}y_{i}\cdot \log\hat{y_{i}}$$

where *N* is the output size, *y* is the target value and $\hat{y}$ is the predicted value.

## 4. Results and Discussion

### 4.1. Data Collection

To the best of our knowledge, there are no publicly available datasets that match the requirements to evaluate the proposed IPS. These requirements include human trajectories containing both inertial and magnetic measurements, along with ground-truth positions of the building’s map for the fingerprint creation. Hence, we created a dataset for the development and evaluation of the presented framework. The data were collected in a floor plan of an office building with 35 m by 12 m. The dataset contains a diversity of human trajectories acquired by different users and smartphones on distinct days to demonstrate the robustness of our framework. The annotation of the trajectories was done for validation purposes.

The data collection was conducted with a logger app that records data from the accelerometer, magnetometer and gyroscope. Additionally, this app allows for the annotation of events by the registry of their timestamps when a user taps the screen.

To ensure consistency between acquisitions, some guidelines were provided to users for the data collection procedure:

- Before starting each trajectory collection, the user was asked to calibrate the smartphone’s magnetometer. The tri-axis magnetometer calibration was guaranteed by performing the movement of drawing a big eight in the air with the smartphone;
- The initial and final positions, together with all direction changes, were annotated on the smartphone, saving the corresponding timestamps. This procedure allowed to create a set of reference points during the acquisition where the real position and the associated magnetic field values are well known;
- The user was asked to maintain a constant velocity during the acquisition so that the distance between annotated positions could be linearly interpolated. We simplified this analysis since this framework will be combined with an existing indoor location solution [16] that already covers the automatic estimation of the distance travelled by the user, with step detection and dead reckoning methods;
- Finally, during the data collection process, the smartphone was being held in front of the user to facilitate the annotation procedure.

### 4.2. Dataset

The created dataset is composed of 27 trajectories across the building, which are split into two groups. The data were collected on different days and times of the day to ensure the variability of a real scenario.

**Figure 7:**
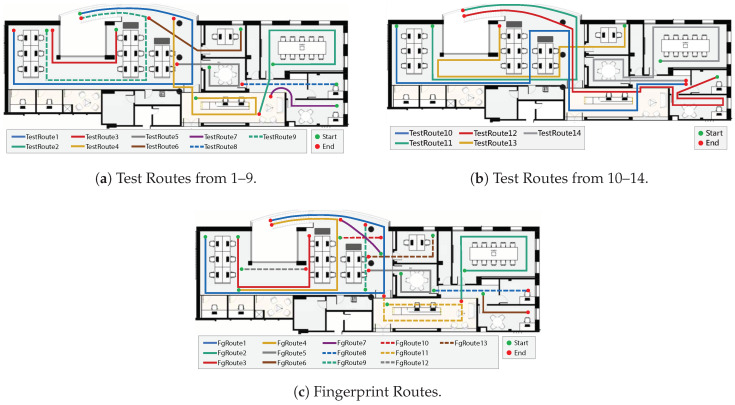
Representation of the dataset routes in different colours and lines. The starting point and endpoint are coloured in green and red, respectively. Routes from (**a**) and (**b**) compose the test set. Routes from (**c**) are used for fingerprinting (acquisitions from user 1) and also for the test set (acquisitions from user 2).

The Fingerprint Routes are available in Figure 7c and were designed to construct the magnetic fingerprint of the building. These routes follow the process of the traditional construction of fingerprints, where a user covers the main halls and rooms of the building, from a start to an endpoint. The criterion was to design the strictly necessary trajectories to optimise as possible the usual time-consuming process. Each corridor should have a single passage, except in large corridors, where more than one trajectory may be designed. Some rooms were left out for their small dimensions.

The Test Routes are depicted in Figure 7a,b and cover the area of the constructed fingerprints to assert the robustness of the proposed framework. These trajectories may overlap with Fingerprint Routes, but some conditions differ, as in some cases the direction of movement. Data were collected by four users with five different smartphones, ranging from older to newer Android versions: LG G7, Xiaomi Mi A2 Lite and Mi8, Huawei Nexus 6P and Google Pixel. The magnetic sensors characteristics of the different devices are presented in Appendix A Table A1. 117 acquisitions were collected, which include a total of 73 min of data. The complete dataset attributes are available in Appendix B Table A2.

#### Train, Validation and Test Sets

All collected data from Fingerprint Routes and Test Routes were preprocessed, as described in Section 3.3. During our experiments, we found out that the z-axis from the magnetic data, together with the computed magnitude, were the components that provided the most valuable information. Therefore, they were the only leveraged components.

**Figure 8:**
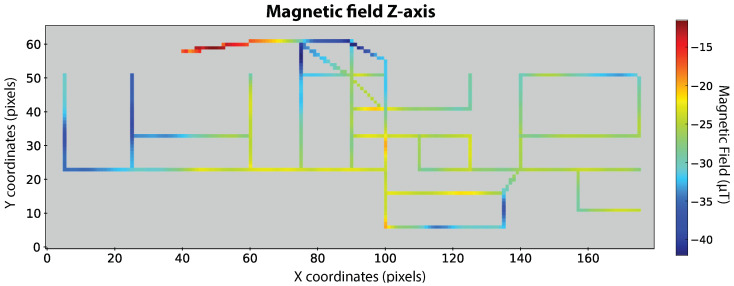
Fingerprint with 0.2 resolution of magnetic field values in *z*-axis.

The fingerprint construction process, described in Section 3.2, was performed using a single set of Fingerprint Routes, collected by user 1 with the LG G7 smartphone. The resulting fingerprint is displayed in Figure 8 and includes the main building corridors and walking paths inside most rooms. It has a resolution of 0.2 m per pixel, which is possible after the linear interpolation of magnetic field values between the annotated coordinates in the designed trajectories, i.e., the initial and final positions, in addition to all direction changes. This results in a set of coordinates with a maximum difference of 0.2 m on both the *x* and *y* axes.

The training set was obtained from the constructed fingerprint, with the data augmentation process of Section 3.4. The length of each sequence L was defined as 4 m, each containing $L=\frac{length\;(in\;meters)}{resolution}=\frac{4}{0.2}=20$ magnetic field values. Followed by the noise addition process, a total of 5688 sequences were created for training the neural network architecture.

Regarding the validation and test sets, the magnetic sequences were extracted from all used acquisitions, following the process of Section 3.3. However, to effectively evaluate the performance close to real scenarios, the noise addition process was not applied. As such, the validation set was constructed using data from Fingerprint Routes of user 2, collected with the Google Pixel. Lastly, the test set contained the Fingerprint Routes of user 2 that were collected with the Xiaomi Mi8, together with the Test Routes from all users with all smartphones. After obtaining the magnetic sequences, the inputs for the neural network were the extracted features from Section 3.5.

### 4.3. Evaluation

After training the model, an experimental evaluation using the test set was performed. Our method follows the common usage of fingerprinting-based IPS, where the most probable regions are used either to provide a user’s location or to at least initialise the localisation procedure. As such, the output of our DNN is a probability map, from which the most probable regions are selected. This allows reducing the localisation search area and might provide further improvement to Wi-Fi dependent multi-source systems, e.g., Wi-Fi-based IPS contain errors varying from 5–15 m [20].

In order to evaluate our results, we defined a new metric, the Success Rate, given by Equation (6), that was applied to the most probable regions from postprocessing.

(6) $$Success\;Rate=\frac{Correct\;Predictions}{All\;Predictions}$$

A Correct Prediction was defined as a prediction that contains at least one probable pixel in a distance to the true position shorter than 4 m. The distance was measured by Dijkstra’s algorithm [21], which finds the shortest path between nodes in a graph, in this case between filled pixels in the probability map. The 4 m value was empirically chosen since it maximises the relationship of Success Rate versus distance to prediction value and it is a value that we consider acceptable for IPS. Our goal was to maximise the Success Rate while reducing the distance error.

Additionally, a distance error between the true position and the closest probable pixel using Dijkstra’s distance for each predicted position was also considered for evaluation purposes.

### 4.4. Postprocessing

In a real case scenario, either for magnetic-based single-source or multi-source localisation systems, it is necessary to determine a set of regions where the user/device may be located. For this purpose, postprocessing was applied to the predicted probability maps to discover the most probable regions.

In this work, we introduce two postprocessing techniques, one based on a threshold and other on the selection of the N-pixels with the highest probability. With the combination of both techniques, the final result is a map containing the most likely regions.

#### 4.4.1. Threshold Approach

**Figure 9:**
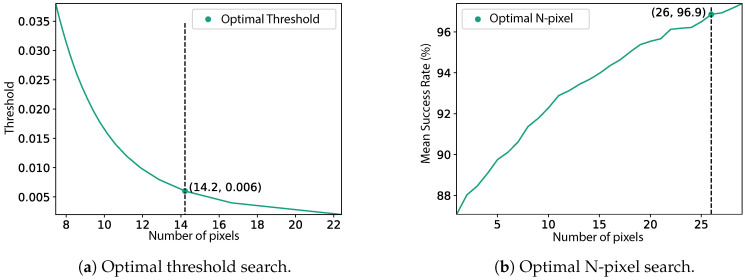
Selection of the optimal threshold and N-pixel value, obtained from the KneeLocator package [22].

In the first approach, we defined a threshold to select the first set of possible regions from the probability map, containing all pixels (i.e., positions) above the select value. This threshold cannot be selected based solely on the improvement of the Success Rate, as in an extreme case, it would be so small that all possible pixels would be included. Therefore, we searched for the threshold the maximises the Success Rate but minimises the number of pixels included. For this purpose, a range of thresholds was tested on the training set (from 0.002 to 0.04). The relationship between the achieved Success Rate and the mean number of included pixels is plotted in Figure 9a. The optimal threshold value corresponds to the knee of the curve, here obtained from the KneeLocator package [22]. The optimal threshold was found to be 0.006, which achieved a Success Rate of 96.4%, with a mean number of pixels included of 14.2 (2.84 m). This means that by only considering the pixels with a probability higher than 0.006, a mean of 14.2 pixels have a probability above the threshold, from which we can achieve a Success Rate of 96.4%.

#### 4.4.2. N-Pixel Approach

In the second approach, from the probability map, the N pixels (i.e., positions) with the highest probability were retrieved. For this purpose, we tested different N pixels values (ranging from 1 pixel (0.2 m) to 30 pixels (6 m)) to assert the value that gives the highest mean Success Rate, from all routes of the training set. The relationship between the number of pixels and the mean Success Rate is represented in Figure 9b. The optimal N, obtained with the KneeLocator package, was 26 pixels (5.2 m), with a Success Rate of 96.9%. In other words, by considering only the 26 pixels with the highest probability we can achieve a Success Rate of 96.9%.

#### 4.4.3. Combination

The threshold approach provided a probability threshold of 0.006, and the N-pixel approach defined an optimal value of 26 best pixels. To evaluate the performance of the DNN, we decided to combine both approaches, considering a maximum of 26 pixels (5.2 m) and a minimum probability of 0.006. With both conditions, we obtained the results of Table 4, which includes the routes from Test Routes (Figure 7a,b) and Fingerprint Routes (Figure 7c) that were not used for training.

### 4.5. Experimental Results

The experimental results for the test set are presented in Table 4. The Success Rate was calculated using Equation (6) and the distance error refers to the mean Dijkstra’s distance within each route from the ground truth position to the closest predicted region. The results demonstrate that the proposed method has a Success Rate of around 80% and a distance error of 2.5 m to the correct position.

Moreover, the results show consistency between different users and smartphones. Hence, it was possible to overcome the difficulty of employing different devices.

Further analysis of the results allows concluding that in all acquisitions of the same designed trajectory, the Success Rate remains similar. For instance, TestRoute10 Success Rate ranges from 69 to 76% and TestRoute13 Success Rate ranges from 82 to 95%.

Some trajectories consistently obtain an excellent classification, as in the case of TestRoute11. By comparing its design in Figure 7b with the magnetic fingerprint of Figure 8, it is visible that this route covers regions with high magnetic field disturbances, as a handrail, which can be seen by the red area. On the other hand, FgRoute6 and FgRoute7 achieved a Success Rate of 0%. From Table A2, we can notice that these two routes correspond to small routes that contain a total of 27 and 33 classified sequences, respectively. Furthermore, by analysing Figure 7a and the magnetic fingerprint in Figure 8, we notice that these regions do not have distinct magnetic disturbances. It is reasonable that our method does not classify them correctly since regions with no magnetic disturbances are more frequent around a building and thus, ambiguous for the DNN model.

**Figure 10:**
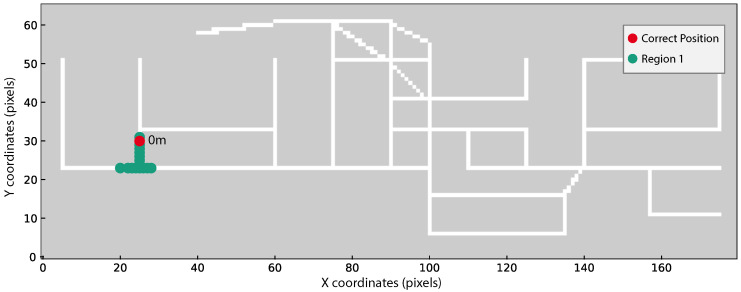
TestRoute11 result from user 4 and Xiaomi Mi A2 Lite. Only one region, in green, is obtained through the combined evaluation methodology. The distance, in meters, from the region to the correct position, marked in red, is provided. This result is considered correct since the green region matches the correct position.

**Figure 11:**
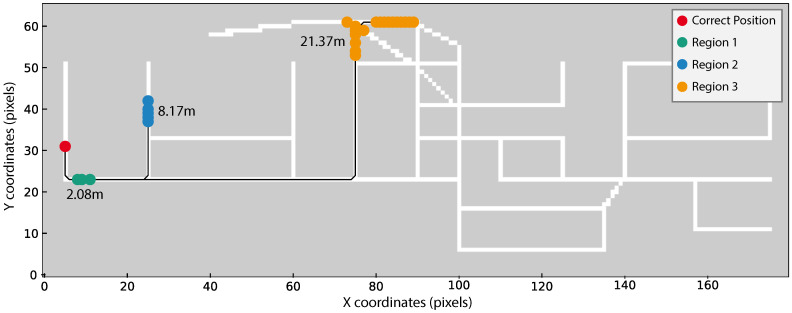
TestRoute10 result from user 4 and Xiaomi Mi A2 Lite. Regions in green, blue and orange, are obtained after applying the combination of the Threshold and N-pixel approaches. Distances, in meters, from each region to the correct position, marked in red, are also depicted. As the green region is less than 4 m way of the correct position this result is considered correct.

**Figure 12:**
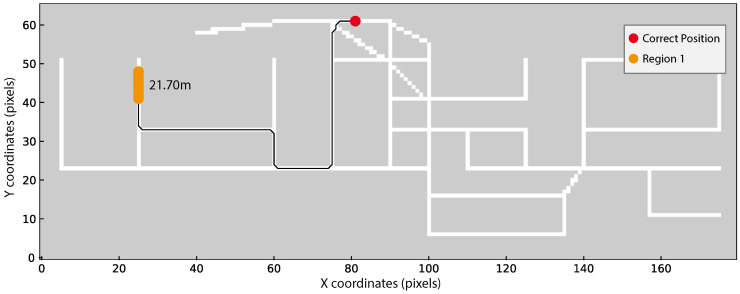
FgRoute1 result from user 2 and Xiaomi Mi8. Incorrect predicted region in orange. Distance, in meters, from the predicted region to the correct position, marked in red, is depicted. This result is considered incorrect since the distance of the correct position to the region is more than 4 m.

As we are using a non-standard evaluation criterion (see Equation (6)), three different scenarios using real acquisitions were selected to better demonstrate the possible position outputs from our method. The three scenarios are the following:

- True position within the obtained regions (Figure 10);
- True position within a maximum distance of 4 m from one of the obtained regions (Figure 11);
- True position beyond 4 m from one of the obtained regions (Figure 12).

In Figure 10, Figure 11 and Figure 12 the obtained regions are depicted in different colours (green, blue, or orange), the correct device position is marked in red and the shorter distance from each region to the true position using Dijkstra’s algorithm is displayed close to each region.

In Figure 10 we can perceive that a single region is obtained, in green, and the true position, in red, is overlaid with the green region. This Figure exhibits the best positioning scenario, where our localisation system only estimates one region containing the correct user position. This result was obtained considering the TestRoute11 outcome from user 4 using the Xiaomi Mi A2 Lite smartphone.

Regarding Figure 11, the combined postprocessing method was applied to TestRoute10 from user 4 while using Xiaomi Mi A2 Lite. This figure highlights three regions depicted in colours green, blue, and orange. Although none of the regions contains the correct position, the green region is within 4 m from the true position. Our algorithms considers this region as correct, since the difference can be associated with errors in the true position estimation. The true position is estimated based on the assumption that users walked in a constant velocity during data acquisition as described in the data collection guidelines (see Section 4.1). However, it is very likely that the velocity changed during the acquisition, compromising the estimation of the true position. Additionally, inaccuracies inherent to the process of manual annotation may also have a negative impact on the true position estimation.

Finally, Figure 12 illustrates the worst scenario where only one region is estimated far from the correct position. In order to understand this result, we can observe the magnetic fingerprint presented in Figure 8 and verify that the magnetic field pattern around the true position and the obtained region are at least similar in the z-axis. Probably, the correct region has also a high probability but it was lower than the probability threshold used. We anticipate that this case shall not occur frequently as we obtained generally good Success Rates (see Table 4).

Regarding the number of the returned regions, ideally our approach should always return only one region, as in the example of Figure 10. However, when a magnetic field sequence shows a similar pattern in various regions of the building, it is expected that more than one region is returned (see Figure 11). This limitation may be reduced by adding a longer magnetic field sequence instead of the used 4 m. However, in this case, the system initialisation will need to wait until the user walked more than 4 m to return the first position. As we previously mentioned, our main motivation is to replace the dependency of RF signals to the system initialisation process. This way, instead of reducing the number of regions we decided to reduce the length of magnetic field sequences. The limitation of obtaining different regions could be tackled by the integration of our methodology with a multi-source localisation system as the one of Guimaraes et al. [16]. Therefore, providing more than one region might not be considered a disadvantage but a strategy to reduce the localisation search to certain areas of the map.

Regarding the possibility of overfitting, due to the lack of data variability while training the model (one user and one smartphone), we believe that it is low after analysing the obtained test results. The test results showed that the model provides satisfactory performance results for different users and smartphones.

## 5. Conclusions

Indoor location offers undoubtedly great potential for a wide range of applications. Most infrastructure-free methods rely only on Wi-Fi signals, and when a combination of multiple data sources is used, systems’ initialisation is frequently dependent on Wi-Fi availability. Nevertheless, there is a need to find alternatives, as Wi-Fi scans are becoming limited in recent Android versions, or not available at all in the case of iOS. Furthermore, the rise of 5G technology may also compromise Wi-Fi usage.

Herewith, we proposed an infrastructure-free IPS, that is user and usage-independent, and only relies on the magnetic field sensing information for the localisation. We designed a dataset for conducting this experiment with different users and smartphones. This dataset was collected in an office building and contains over 117 acquisitions, fulfilling a total of 73 min of data. With data from one user and one smartphone, we created a fingerprint of the magnetic field values. Moreover, to produce a sufficiently large training dataset, we developed a data augmentation algorithm based on the created fingerprint.

To better describe existing magnetic patterns inside buildings, significant features were extracted from the training data. Afterwards, an architecture with four DNNs was fed with the absolute magnetic field and the extracted features. The implemented DNN provides, as output, a probability map of the user’s location, which then undergoes two postprocessing methods, resulting in the most probable location regions.

After applying the postprocessing methods, we achieved considerably good results since the acquisitions Success Rate is about 80%, within a 4 m distance, and the average distance error corresponds to 2.5 m. Considering that we used mainly magnetic data, the results support the potential of this source to provide complementary information for current IPS.

The magnetic field as a single source is unreliable for some applications, as it is easily affected by temporary interferences. Still, most multi-source IPS that leverage the magnetic field do not fully explore its potentialities. Thus, with this work, we aimed to study how this source can complement other solutions.

Comparing to existing IPS, our approach leverages the DNN capabilities by presenting an alternative to typical Wi-Fi based methodologies obtaining satisfactory positioning results. Although our solution can be used as an independent indoor location system, we believe that its main potential is in the integration with other multi-source systems, during the initialisation phase as an alternative to RF signals. In future work, the integration of our approach with the work of Guimaraes [16] will be addressed, where our framework will be introduced as a new layer for the fused positioning mechanism and for initialisation purposes.

Due to the magnetic field dependency of our method, we envisage a challenge regarding the possible changes of buildings environmental characteristics, which can create different magnetic field patterns, requiring recurrent data collections to remap the fingerprints. However, using crowdsourcing techniques for fingerprints updates can mitigate this limitation. In addition, since magnetometer sensors can easily decalibrate, our system would need an automatic magnetometer calibration method to avoid inconsistent location previsions.

In future directions, our methodology could be employed alongside other data sources, thus, providing a more robust system. In situations in which there is more than one high probability region, the combination of different data can provide a straightforward localisation response. Additionally, by providing the most likely regions, the magnetic field data can also help with the initialisation of other systems. Finally, to test the effectiveness and robustness of our methodology, we will employ it in different locations.

## Figures and Tables

**Table 1:** Deep Neural Networks (DNN) Type 1 layers’ details, including the type of layer, the number of hidden units, the kernel size and the activation function. The kernel size of three was employed in the magnetic differences feature set, while the kernel size of five was used for the combinatorial magnetic differences set.

Layer	Hidden Units	Kernel Size	Activation Function
Conv1D	16	3 (5)	ReLu
Conv1D	32	3 (5)	ReLu
Conv1D	64	3 (5)	ReLu
Conv1D	256	3 (5)	ReLu
MaxPooling1D	-	-	-
Dense	512	-	Linear

**Table 2:** DNN Type 2 layers’ details, including the type of layer, the number of hidden units and the activation function. This architecture was used with the absolute magnetic field values.

Layer	Hidden Units	Activation Function
LSTM	16	Tanh
LSTM	32	Tanh
LSTM	64	Tanh
LSTM	256	Tanh
Dense	512	Linear

**Table 3:** Neural Network Combination. Layer details, including type of layer, hidden units, activation function and output shape. This neural network combined the outcomes of the three implement networks, two of type 1 and one of type 2.

Layer	Hidden Units	Activation Function	Output Shape
Dense	11,880	Softmax	-
Reshape	-	-	(66, 180)

**Table 4:** Results for the test set, including Test Routes 1-14 and Fingerprint Routes. Smartphones A–E are respectively LGE LM-G710, Mi A2 Lite, Mi8, Nexus 6P and Pixel.

Acquisition	User	Smartphone	Success Rate (%)	Distance Error (m)	Acquisition	User	Smartphone	Success Rate (%)	Distance Error (m)
TestRoute1	2	E	84	1.77	TestRoute12	2	C	69	4.30
TestRoute1	1	A	83	1.53	TestRoute12	2	E	74	3.87
TestRoute2	2	E	93	1.05	TestRoute12	2	B	69	4.47
TestRoute2	1	A	100	0.03	TestRoute12	3	D	65	4.60
TestRoute3	2	E	100	0.59	TestRoute12	1	A	74	3.56
TestRoute3	1	A	88	1.30	TestRoute13	4	B	89	2.00
TestRoute4	2	E	59	3.64	TestRoute13	2	C	83	3.03
TestRoute4	1	A	70	2.54	TestRoute13	2	E	95	1.43
TestRoute5	2	E	36	4.80	TestRoute13	2	B	82	3.07
TestRoute5	1	A	77	2.82	TestRoute13	3	D	87	2.62
TestRoute6	2	E	63	3.63	TestRoute13	1	A	87	2.18
TestRoute6	1	A	90	1.69	TestRoute14	4	B	90	1.19
TestRoute7	2	E	87	1.36	TestRoute14	2	C	85	2.44
TestRoute7	1	A	80	1.70	TestRoute14	2	E	79	2.48
TestRoute8	2	E	100	0.00	TestRoute14	2	B	76	3.32
TestRoute8	1	A	100	0.28	TestRoute14	3	D	84	1.54
TestRoute9	2	E	81	3.39	TestRoute14	1	A	82	2.10
TestRoute9	1	A	87	2.74	FgRoute1	2	C	99	0.36
TestRoute10	4	B	69	3.74	FgRoute2	2	C	82	4.13
TestRoute10	2	C	75	3.25	FgRoute3	2	C	87	1.82
TestRoute10	2	E	74	3.27	FgRoute4	2	C	89	1.45
TestRoute10	2	B	76	3.16	FgRoute5	2	C	49	4.98
TestRoute10	3	D	64	4.24	FgRoute6	2	C	0	8.75
TestRoute10	1	A	76	2.83	FgRoute7	2	C	0	11.17
TestRoute11	4	B	100	0.80	FgRoute8	2	C	97	0.59
TestRoute11	2	C	95	1.10	FgRoute9	2	C	100	0.00
TestRoute11	2	E	97	1.33	FgRoute10	2	C	100	0.00
TestRoute11	2	B	92	2.01	FgRoute11	2	C	77	1.72
TestRoute11	3	D	91	1.40	FgRoute12	2	C	100	0.00
TestRoute11	1	A	93	1.47	FgRoute13	2	C	88	1.90
TestRoute12	4	B	70	4.28					
**Average Success Rate ($\bar{\mu }\pm \sigma$)** 80 ± 20									
**Average Distance Error ($\bar{\mu }\pm \sigma$)** 2.50 ± 1.93									

## Abbreviations

The following abbreviations are used in this manuscript:

IPS	Indoor Positioning System
DNN	Deep Neural Networks
GNSS	Global Navigation Satellite System
RFID	Radio Frequency Identification
UWB	Ultra-wideband
WAP	Wireless Access Points
RF	Radio Frequency
MEMS	Micro-Electromechanical System
MP	Magnetic Patterns
DTW	Dynamic Time Warping
LSTM	Long-Short-Term-Memory
CNN	Convolutional Neural Network
RNN	Recurrent Neural Network

## Appendix A. Technical Characteristics of Magnetic Smartphone Sensors

Table A1 contains the main technical characteristics of each magnetic smartphone sensor used in this study.

**Table A1:** Technical characteristics of each magnetic smartphone sensor including magnetometer type, sensing range, sensitivity and resolution.

Smartphone	Magnetometer	Sensing Range (mT)	Sensitivity (μT/LSB)	Resolution (bites)
LGE LM-G710	AKM AK09918C ${}^{1}$	±4.9	0.15	16
Xiaomi Mi A2 Lite	AKM AK09918C ${}^{1}$	±4.9	0.15	16
Xiaomi Mi8	AKM AK09918C ${}^{1}$	±4.9	0.15	16
Nexus 6P	BMM150 ${}^{2}$	±1.3 (X, Y), ±2.5 (Z)	-	13
Google Pixel	AKM AK09915C ${}^{1}$	4.7∼5.2	0.15	16

${}^{1}$https://www.akm.com/kr/ko/products/electronic-compass/specifications-comparison/; ${}^{2}$https://www.bosch-sensortec.com/products/motion-sensors/magnetometers-bmm150/.

## Appendix B. Dataset Description

Table A2 contains the details of the dataset collected for this work. The dataset was collected by different users and smartphones, and it is composed by trajectories acquired for the fingerprint creation, FgRoute, and for testing purposes, TestRoute.

**Table A2:** Dataset and their characteristics including the number of acquisitions and instances, users that participated in each acquisition and smartphones used. The number of instances corresponds to the total number of labels in each trajectory. Smartphones A, B, C, D and E are respectively LGE LM-G710, Mi A2 Lite, Mi8, Nexus 6P, Pixel.

Trajectory	# Acquisitions	# Instances	User	Smartphone
TestRoute1	2	400	1, 2	A, E
TestRoute2	2	216	1, 2	A, E
TestRoute3	2	112	1, 2	A, E
TestRoute4	2	256	1, 2	A, E
TestRoute5	2	106	1, 2	A, E
TestRoute6	2	82	1, 2	A, E
TestRoute7	2	30	1, 2	A, E
TestRoute8	2	58	1, 2	A, E
TestRoute9	2	270	1, 2	A, E
TestRoute10	12	6288	1, 2, 3, 4	A, B, C, D, E
TestRoute11	12	4368	1, 2, 3, 4	A, B, C, D, E
TestRoute12	12	5544	1, 2, 3, 4	A, B, C, D, E
TestRoute13	12	4440	1, 2, 3, 4	A, B, C, D, E
TestRoute14	12	4488	1, 2, 3, 4	A, B, C, D, E
FgRoute1	3	579	1, 2	A, C, E
FgRoute2	3	339	1, 2	A, C, E
FgRoute3	3	207	1, 2	A, C, E
FgRoute4	3	306	1, 2	A, C, E
FgRoute5	3	147	1, 2	A, C, E
FgRoute6	3	27	1, 2	A, C, E
FgRoute7	3	33	1, 2	A, C, E
FgRoute8	3	87	1, 2	A, C, E
FgRoute9	3	51	1, 2	A, C, E
FgRoute10	3	12	1, 2	A, C, E
FgRoute11	3	231	1, 2	A, C, E
FgRoute12	3	42	1, 2	A, C, E
FgRoute13	3	73	1, 2	A, C, E
**Total:** 27	117	28,792	4	5
